# Challenges and Recommendations for Oral Healthcare of Older Adults in a Long-Term Care Facility

**DOI:** 10.3390/healthcare13202642

**Published:** 2025-10-20

**Authors:** Haslina Rani, Amalina Alya Azizan, Nurul Izzah Abdul Walad, Siti Aisya Athirah Hassan, Tuti Ningseh Mohd Dom, Daphne Shu Huey Yeoh, Joyce Wuen Cheer Tay, Muhammad Syafiq Asyraf Rosli, Nur Saadah Mohamad Aun, Aznida Firzah Abdul Aziz, Kaung Myat Thwin, In Meei Tew

**Affiliations:** 1 Faculty of Dentistry, University Kebangsaan Malaysia (UKM), Jalan Raja Muda Abdul Aziz, Kuala Lumpur 50300, Malaysia; hr@ukm.edu.my (H.R.); a182917@siswa.ukm.edu.my (A.A.A.); a182896@siswa.ukm.edu.my (N.I.A.W.); tutinin@ukm.edu.my (T.N.M.D.); a182269@siswa.ukm.edu.my (D.S.H.Y.); a182294@siswa.ukm.edu.my (J.W.C.T.); syafiqasyraf@ukm.edu.my (M.S.A.R.); 2 Faculty of Social Sciences and Humanities, University Kebangsaan Malaysia (UKM), Bangi 43600, Selangor, Malaysia; p144770@siswa.ukm.edu.my (S.A.A.H.); n_saadah@ukm.edu.my (N.S.M.A.); 3 Faculty of Medicine, Universiti Kebangsaan Malaysia (UKM), Cheras, Kuala Lumpur 56000, Malaysia; draznida@hctm.ukm.edu.my; 4 Faculty of Dentistry, Niigata University, Niigata 951-8514, Japan; kaung@dent.niigata-u.ac.jp

**Keywords:** barriers, domiciliary care, older adults, dental health

## Abstract

**Background/Objectives:** As the aging population is growing globally, oral health has become integral to ensuring healthy aging and quality of life. This study assessed the oral health status of older adults in a Malaysian long-term care facility and explored caregiver-reported challenges in providing oral care. **Methods:** A convergent mixed-methods design was applied, involving 115 residents aged ≥60 years and 16 caregivers in a public facility. The residents’ oral health was assessed using interviewer-assisted questionnaires (demography, dependency level, Oral Frailty Five-item Checklist), clinical examinations (dental caries status, number of remaining teeth, oral and denture hygiene), and the Decayed, Missing, and Filled Teeth (DMFT) index. Focus group discussions with caregivers were conducted, transcribed, and thematically analyzed. Quantitative data were descriptively analyzed using SPSS version 29.0. **Results:** Over one-third of the residents (39%) were moderately to highly dependent on caregivers. All had experienced dental caries, with most having fewer than 20 teeth (92.9%) and requiring dentures (81.7%). Overall, both oral and denture hygiene were poor. Assessment of oral frailty indicated that the majority of residents (94.8%) were at risk of impaired oral function. A thematic analysis identified four key themes influencing oral health: (1) health and oral health conditions of residents; (2) variety in oral care practices; (3) older adults’ attitudes and behaviors; and (4) system factors. These themes were mapped in a conceptual framework demonstrating multilevel influences on oral care. **Conclusions:** Despite the single-center design, these findings provide actionable insights for improving geriatric oral health policies in Malaysia. Practical recommendations include integrating oral health into aged-care standards, expanding mobile dental services, and establishing oral care champions within facilities. Addressing these challenges is critical to improving quality of life and aligning long-term care practices with the WHO’s healthy aging priorities.

## 1. Introduction

The world is undergoing a demographic transition, with older adults comprising an increasing share of the population. In this study, “older adults” are defined as individuals aged ≥60 years, consistent with the WHO’s usage of the term. Global projections, however, often report figures for those aged 65 years and above; for instance, it is estimated that one in six people will be over the age of 65 by 2050, putting tremendous pressure on healthcare systems [1].

With increasing life expectancy, the focus of healthcare has shifted from merely extending life to improving its quality. However, aging is often accompanied by chronic conditions such as diabetes, cardiovascular diseases, and neurodegenerative disorders [2,3], which may compromise oral health. Poor oral health among older adults is associated with malnutrition [4], aspiration pneumonia [5], and reduced quality of life [6]. Global policies, such as the WHO Global Strategy and Action Plan on Oral Health 2023–2030, now emphasize integrating oral health within primary and long-term care systems [7,8].

In Malaysia, the National Oral Health Survey of Adults (NOHSA) 2010 reported that 98.3% of dentate older adults exhibited signs of periodontal disease, while 76.7% were fully edentulous [9]. Despite these alarming statistics, oral healthcare remains a neglected component of geriatric care, particularly in long-term care facilities (LTCFs). This issue is expected to worsen as the nation ages, with 16% of Malaysians projected to be ≥65 years by 2050 [10].

Residents of LTCFs often struggle to maintain oral hygiene because of physical limitations, cognitive impairments, and reliance on caregivers [11]. International and local studies have shown that caregivers frequently lack the necessary knowledge and skills for oral hygiene provision [12,13]. Collectively, these findings mirror global reports of persistent implementation gaps, limited workforce capacity, and inconsistent care protocols in LTCFs [14].

Access to professional dental treatment presents another major challenge. Conventional dental services are often not designed to accommodate for the complex medical and physical needs of frail older adults, many of whom face difficulties in traveling to dental clinics. Domiciliary oral care services, where dentists visit long-term care facilities to treat patients, offer a potential solution. However, such services are sparse in Malaysia because of systemic, financial, and logistical barriers including a shortage of qualified geriatric dentists and a lack of funds for mobile dental units [15]. Without coordinated and sustained interventions, these challenges will continue to compromise the oral health of nursing home residents, ultimately affecting their overall health and quality of life. In line with these challenges, the WHO 2023–2030 agenda and healthy aging guidance (ICOPE) advocate for person-centered pathways and service models that embed essential oral care into primary and long-term care, reinforcing the relevance of domiciliary approaches for frail, dependent older adults [14].

Although research on geriatric oral health is growing, few studies have specifically addressed institutionalized older people and the role of caregivers in maintaining oral hygiene. As the primary providers of daily dental care in long-term care facilities, caregivers’ perspectives are essential for identifying gaps in knowledge, resources, and systemic support. Understanding the challenges faced by caregivers in providing oral healthcare is particularly important given Malaysia’s rapidly aging population and the growing number of older adults residing in LTCFs.

While global data highlight the importance of oral health in aging, evidence from institutionalized Malaysian older adults remains limited. By aligning with the current WHO priorities and translating them to the Malaysian LTC context, this study responds directly to the global call to close evidence-to-practice gaps for institutionalized older adults and enhance the geographic context and regional relevance. Therefore, the purpose of this study was to assess the oral health status of residents in a public long-term care facility and to identify the challenges associated with providing oral care to the residents from the caregivers’ perspectives.

## 2. Materials and Methods

### 2.1. Study Design

In this convergent quantitative–qualitative study, two independent studies were conducted and then the end results were merged to collate a comprehensive understanding of the research problem. An interviewer-assisted questionnaire with a clinical examination was conducted to assess the oral health status of the older adults, while a series of focus group discussions were conducted among the caregivers to identify the challenges related to providing oral care in a long-term care facility.

### 2.2. Sampling Strategy

This study was conducted at a single public long-term care facility. This facility was selected based on its large resident population and strong administrative support. The facility houses approximately 200 older adults and employs over 30 caregivers. It has access to government-funded healthcare, including oral health services. The study participants were recruited using a purposive sampling technique with the help of the unit managers within the facility. The inclusion criteria for the older adults were that they had to be at least 60 years old and Malaysian. Those with cognitive issues and unable to understand or provide consent to participate in the study were excluded. Cognitive ability was assessed informally during recruitment by determining whether the participant could understand simple instructions, follow basic conversation, and demonstrate adequate comprehension to provide informed consent. The sample size estimated for the sampling of older adults in this study was 120. This number was calculated with a 5% margin of error and confidence level of 95%, based on an 8.5% prevalence of frailty among Malaysians aged 60 years and above in Malaysia [16].

The included caregivers were at least 18 years old, spoke Malay or English, and worked full-time or part-time at the nursing home. All caregivers were health attendants with the same caregiving role, which was to care for the daily living needs of the older adults at the facility. Caregivers with less than one year of experience in older adults’ care were excluded from the study. The data collection continued until data saturation was reached, defined as the point at which no new themes or information emerged, and all the topics in the interview guide were sufficiently explored.

### 2.3. Ethics

We obtained ethical approval for this study from the Research Ethics Committee of Universiti Kebangsaan Malaysia (JEP-2024-543). Permission to conduct the study was also granted by the Malaysian Department of Social Welfare. All participants provided written informed consent after being briefed on the study’s purpose and procedures.

### 2.4. Data Collection Methods

#### 2.4.1. Questionnaire and Clinical Assessments of Older Adults

This study was conducted throughout November 2024. The data collection tools for older adults were divided into three main parts: A, B, and C. Part A consisted of obtaining basic information about the participants, such as age, race, gender, and dependency level, assessed using the Canadian Study of Health and Aging (CSHA) framework [17].

Part B assessed oral frailty using the Oral Frailty Five-item checklist, a tool used to assess oral function in older adults [18]. The tool evaluates five key aspects of oral health: chewing difficulties, swallowing difficulties, and dry mouth were assessed via interviews; the number of remaining teeth was confirmed through clinical examination; and articulatory motor skills was evaluated using oral diadochokinesis, where the participants were asked to repetitively articulate the syllable “ta” as quickly as possible for 5 s. The number of “ta” sounds produced was counted using the dot method and recorded. Participants who produced fewer than 6 “ta” sounds per second were categorized as having low articulatory oral motor skills. A participant was considered to have a risk of poor oral function if they met two or more of the above criteria.

The researchers interviewed the participants to answer all the questions in Part A and three questions in part B. The questions were asked in colloquial, daily conversational language, which is a natural mix of Malay and Mandarin commonly used in the facility that ensured that the residents could easily understand them.

Part C of the form recorded the participants’ clinical oral health status, which comprised dental caries status, number of remaining teeth, plaque control record, denture use, and denture hygiene. Experience with dental caries was assessed using the Decayed, Missing, and Filled Teeth (DMFT) index following the WHO criteria for oral health surveys. Trained examiners conducted clinical oral examinations using a mouth mirror and dental probe under portable lighting. The numbers of teeth with untreated caries (D), teeth missing due to caries (M), and teeth filled due to caries (F) were recorded for each participant. The total DMFT score was calculated as the sum of these components, with a possible range of 0 to 28 [19].

O’Leary’s plaque control record (PCR) was used to evaluate the presence of plaque on the tooth surfaces of the older adults [20]. In PCR, the dental plaque in the oral cavity is stained using plaque-disclosing solutions, and the presence of the plaque is confirmed visually. Each tooth surface is then divided into four blocks (Mesial, Distal, Buccal, and Lingual). The percentage of the plaque score is calculated by dividing the total number of surfaces stained by the total number of tooth surfaces.

Denture hygiene was assessed through direct clinical examination using a scoring system adapted from Mylonas et al. (2014) [21]. A liquid plaque disclosing dye was applied to the fit surface of the denture, which was then visually inspected to determine a denture cleanliness index (DCI) score. The assessment focused only on the fit surface, as this is both the simplest surface to apply the dye and the area most likely to accumulate plaque. The DCI ranges from 0 to 4, with higher scores indicating poorer hygiene: a score of 0 represents a clean denture with no visible plaque, staining, or detectable deposits; a score of 1 indicates a visibly clean denture with minimal staining affecting less than 25% of the fit surface; a score of 2 denotes visible plaque or debris with moderate staining of 25–50% of the surface; and a score of 3 reflects severe staining with more than 50% of the fit surface affected. A score of 4 is assigned when visible calculus deposits are present on any surface of the denture. In addition, an asterisk (*) is used to denote visible defects in the denture that may act as plaque-retentive sites or require repair or replacement, in conjunction with any of the above scores.

Data collection was piloted with 10 participants to standardize the two researchers performing the dental examinations. Their clinical findings were compared with those of a Prosthodontist who served as the reference examiner. Intra-examiner reliability was assessed through two repeated examinations of the same participants. Inter- and intra-examiner agreement was calculated using Cohen’s kappa.

#### 2.4.2. Focus Group Discussions (FGDs)

A series of focus group discussions (FGDs) were conducted to explore the challenges faced by caregivers in providing oral care to older adults. Five to six participants were recruited for each round based on their availability at the scheduled time. In total, three rounds of FGDs were carried out until data saturation was reached. Prior to the FGD, the caregivers were asked to provide their age, gender, and years of experience in caregiving via a self-administered questionnaire.

Each focus group discussion was facilitated by a trained moderator and supported by a note-taker, neither of whom was affiliated with the caregivers’ workplace. Transcripts were independently coded by two researchers, and differences in interpretation were resolved through iterative team discussions and reference to the raw data until consensus was reached. Although no formal statistical measure of intercoder reliability was applied, the use of independent coding, peer debriefing, and consensus-building enhanced analytic rigor. Reflexivity was maintained by employing trained moderators, maintaining detailed field notes, and conducting regular team debriefings to minimize potential researcher bias.

A semi-structured topic guide was developed based on a review of the relevant literature, current guidelines on geriatric oral health, and input from experts in dental public health and geriatric care. The guide was pilot-tested prior to data collection to ensure that the questions were clear, relevant, and appropriate. Each FGD session lasted for around 45 min to one hour. The moderator initiated each with an open-ended question, prompting the participants to share their perspectives on providing oral care to older residents. The participants were encouraged to contribute freely, and the discussion proceeded sequentially through the topic guide. Probing questions were used to explore emerging issues more deeply or to clarify participant responses. The moderator also ensured balanced participation by encouraging quieter participants to share their views. Field notes were taken to complement the audio recordings.

### 2.5. Data Processing

Quantitative data processing was performed halfway through the data collection visits via SPSS data entry and cleaning. This is to keep track of possible data loss. The process was repeated at the end of the data collection process. For the qualitative data, the processing began immediately after the completion of each of the FGD sessions. The audio recordings were transcribed manually into a written text. The complete transcript was then compared with the handwritten notes taken by the note-taker to verify the data’s accuracy. The transcripts were verified by peer debriefings, where an expert analyzed the data and validated the interpretations of the documentation to avoid possible bias in the data gathering and analyses. To maintain confidentiality, all identifying information (e.g., names and specific locations) was removed, and pseudonyms were assigned to the participants.

### 2.6. Data Analyses and Rigor

The quantitative data were descriptively analyzed in terms of their frequency and mean value. For qualitative data, thematic analysis was inductively conducted on the transcripts to code relevant data. Open coding was performed independently by two researchers to label meaningful units relevant to the research questions by using Delve Qualitative Data Analysis software [22,23]. Delve was selected for its intuitive design and features, which facilitated independent coding by multiple researchers and iterative comparison of codes. As the aim of this study was to perform a thematic analysis rather than advanced query-based analysis, Delve provided sufficient functionality for data management and coding.

The initial codes were reviewed by the research team, and the feedback was used to modify and develop new codes. The coded data were then sorted into potential recurring themes covering the research questions. Thematic saturation was achieved after three FGDs, when no new codes or categories emerged, and additional sessions were unlikely to contribute new insights. The research team met to discuss and reach consensus by reviewing, modifying, and making final refinements to the themes. While no formal intercoder reliability statistics were applied, independent coding, peer debriefing, and consensus-building enhanced rigor. The final data were translated into English and proofread by researchers well-versed in both English and Malay. To increase readability, we removed pauses and words that were not integral to the meaning of the data.

## 3. Results

### 3.1. Characteristics of Older Adults and Demographics of Caregivers

**Figure 1:**
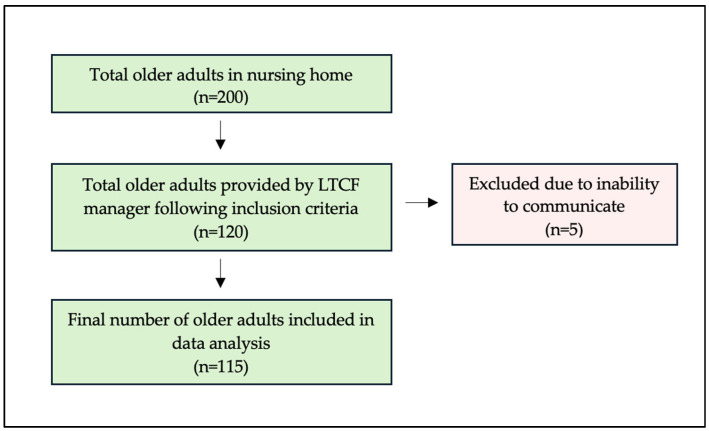
Participant recruitment flow chart.

**Figure 2:**
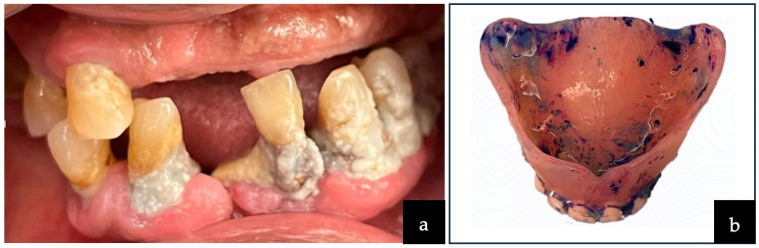
(**a**) Plaque accumulation on all tooth surfaces; (**b**) denture cleanliness index score of 2, indicating 25–50% of visible plaque and debris on denture’s fitting surface.

A total of 115 older adult residents who met the inclusion criteria were included in the study for data analysis (Figure 1). Although the final number of older adult participants was slightly lower than the initially estimated sample size, a post hoc power analysis confirmed that the achieved sample retained an acceptable level of statistical power (β = 0.87) for descriptive purposes. More than one-third (39%) of the older adults had either a medium or high level of dependency on caregivers, and 109 (94.8%) had a risk of poor oral functions. All residents experienced tooth loss, and a large proportion of the residents (81.7%) needed dentures. The overall DMFT index was correspondingly high, with a mean of 25.70 ± 4.30, indicating that the number of missing teeth (24.50 ± 6.40) was considerably higher than untreated decayed (1.0 ± 2.4) and filled teeth (0.30 ± 1.0) (Table 1). Oral and denture hygiene were also found to be poor (Figure 2a,b). The inter-examiner reliability demonstrated almost perfect agreement, with κ = 0.80 (95% CI, 0.70–0.89) for researcher D.S.H.Y. and κ = 0.88 (95% CI, 0.77–0.94) for researcher J.W.C.T., while the intra-examiner reliability showed substantial to almost perfect agreement, with κ = 0.84 (95% CI, 0.72–0.92) for researcher D.S.H.Y. and κ = 0.79 (95% CI, 0.66–0.88) for researcher J.W.C.T.

Table 2 describes the caregivers involved in this study. The mean age of the caregivers was 30.6 (S.D. 8.2) years old. The years of experience varied from 1 to 17 years, with a mean of 5.7 years (Table 2).

The thematic analysis of the qualitative data revealed four themes, and each theme consists of two to five subthemes, which are summarized in Table 3. The themes that emerged in terms of challenges were health and oral health conditions of older adults; variety in oral care practices; older adults’ attitudes and behaviors; and system factors.

**Figure 3:**
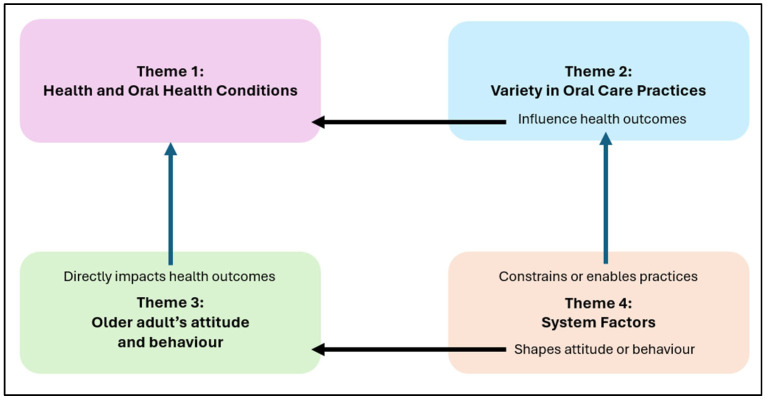
Conceptual framework of caregiver-identified themes.

To aid interpretation, a conceptual framework was developed to illustrate the relationships between these themes (Figure 3). System factors were described by caregivers as shaping both oral care practices and residents’ health conditions while also influencing attitudes and behaviors toward oral care. Caregivers reported that residents’ attitudes and behaviors (e.g., non-cooperation, resistance) directly affected oral health outcomes. Similarly, the variety of oral care practices adopted in the facility was found to influence residents’ oral health conditions. Together, these interconnected themes highlight the multilevel challenges that shape the oral health of older adults in long-term care facilities.

### 3.2. Theme 1: Health and Oral Health Conditions of Older Adults

The caregivers noticed a variety of oral health problems, such as neglected denture care, gum disease, and poor oral hygiene that were described by the caregivers as yellow or black teeth and plaque buildup.

“Their teeth are yellow, plaque-covered, and black. I’ve also seen some that are broken, leaving only stumps.”

[P10]

One of the caregivers also noticed that the older adults in this facility have bad breath, which is one of the signs of poor oral hygiene.

“I often notice that the residents have bad breath.”

[P6]

The caregivers also shared that poor denture hygiene was a recurring issue:

“I saw dentures not cleaned for days, they smell when you take them out.”

[P12]

The caregivers also stated that most of the residents have lost most or all of their teeth, which impacts their masticatory function. They shared that the residents were unable to eat hard food and took longer to eat. Due to this, many residents need dentures to replace their missing teeth to improve their masticatory function.

“Because they have no teeth, it’s difficult for them to eat especially hard food, and it takes longer.”

[P11]

“Some have only root stumps left, no proper teeth anymore, so they just swallow soft food without chewing.”

[P9]

Oral pain and discomfort is a subtheme that describes the challenges of the caregivers in managing complaints of oral pain by the residents. They shared their attempts to alleviate the pain and reduce discomfort by administering painkillers and ice packs while the residents waited to receive professional dental care.

“Painkillers are given first for those with complaints of toothaches.”

[P8]

Moreover, the caregivers in this study said that changing the residents’ food to soft food, such as porridge, is also one of the initiatives performed so that residents can eat more adequately despite experiencing oral pain and discomfort.

“As an alternative when they have toothaches, we give porridge.”

[P5]

In addition, since some older people living within the community have complex medical conditions, it is difficult for the caregiver to ensure that the residents maintain their oral hygiene. They shared that older adults who have complex medical conditions, particularly those who rely on feeding tubes, often have limited mobility, making it hard for the caregivers to help them with their oral health as they often have difficulty opening their mouths. Thus, the caregiver must be creative to navigate these physical disabilities to perform their oral care.

“They have to drink milk through a tube, so it’s hard to open their mouth.”

[P2]

### 3.3. Theme 2: Variety in Oral Care Practices

The caregivers shared the variety of oral care practices performed in this nursing home, which vary based on the resident’s level of independence. Many residents, particularly those who are healthy and independent, are able to perform basic oral hygiene tasks such as brushing their teeth and rinsing their mouths after meals. Furthermore, caregivers also shared the practices related to denture care for residents who use dentures, as stated below.

“Those who are healthy take care of themselves, they can remove and clean their dentures on their own.”

[P14]

However, older adults who are bedridden or have physical disabilities require assisted oral care. The caregivers shared that they assist those who have physical disabilities with their oral care by helping them brush their teeth and clean their gums and tongue with a wet towel.

“We manage them 100%, so sometimes while bathing them, we use a towel to clean their tongue or mouth. For the bedridden ones who drink milk, sometimes we even scrape their mouths.”

[P13]

### 3.4. Theme 3: Older Adults’ Attitudes and Behaviors

In this theme, the caregivers expressed that the residents’ attitudes and behaviors impact the services of oral care provided. Caregivers often face a lack of cooperation from residents when trying to help them with their dental care; many are unwilling to brush their teeth or open their mouths and follow instructions, making the task of providing even the most basic oral hygiene care challenging for caregivers.

“They refuse to cooperate when we try to clean their mouths.”

[P14]

“Sometimes they clamp their mouths shut and refuse to open, even when we explain gently.”

[P9]

The caregivers also expressed that the older adults feel superior to them as they are much older than the caregivers. The residents perceive receiving guidance from predominantly younger caregivers as a challenge to their autonomy, as they believe that they can make independent decisions. Caregivers, in turn, reported experiencing frustration due to difficulties in fulfilling their responsibilities while respecting the preferences of older adults.

“We can’t give them advice or instructions because they feel they are older than us, so we just have to follow their way.”

[P16]

Some of the caregivers shared that some residents were aggressive towards them by scolding and spitting at them when they attempted to provide their oral hygiene care, particularly those who have physical disabilities. This type of behavior makes it difficult for the caregivers to help them perform their oral healthcare.

“When we call the bedridden residents to brush their teeth, they get angry and say it hurts. So how can we brush them?”

[P5]

“One patient spat at me when I tried to brush, so I just stopped.”

[P2]

Caregivers also experienced difficulties related to smoking and vaping that made it even more challenging to provide good oral health among residents who were already reluctant to cooperate with dental care.

“They smoke a lot, not just cigarettes, even vaping is common now. They buy it themselves during their weekly outing”

[P4]

### 3.5. Theme 4: System Factor

This theme reflects the organizational aspects that influence oral care delivery at a long-term care facility. Caregivers stated that they did not have the ability to focus on residents’ oral healthcare due to a lack of manpower. Obviously, this makes it very difficult to put oral care into practice for the residents as it is not their main priority compared to other tasks such as bathing and preparing meals.

“There isn’t enough time to ensure they brush their teeth because we also have to manage meals, due to the lack of staff.”

[P3]

A major system factor identified by caregivers was lack of training, which resulted in poor knowledge or skills about how to deliver oral care, especially in older adults with complex needs. This gap limited the caregivers’ ability to provide effective oral care, resulting in a lack of consistent oral care practices and residents’ oral health needs remaining unmet. Caregivers also expressed that the knowledge they used to provide oral care to the older adults is through experience or basic knowledge, which may not be sufficient to meet the variety of oral health needs of residents.

“I don’t think we’ve received any training or education on oral care especially for the bedridden residents.”

[P15]

“I didn’t receive any training, maybe just from school where they taught basic tooth brushing techniques. Nothing that is specific for older adults.”

[P7]

The caregivers expressed their uncertainty about the correct denture care practices to help the residents take care of their dentures. Some of the caregivers are aware of the need to remove their dentures before bed and soak them overnight; however, they are uncertain about the proper use of denture cleaning solution and general denture maintenance.

“No, because dentures need to be soaked before bed, but the problem is we don’t know the proper way to use the cleaning solution.”

[P3]

“We don’t know the right way to use the denture cleaning liquid, so sometimes we just use water.”

[P3]

While insufficient knowledge, skills, and formal training among caregivers represented a considerable obstacle, additional challenges included restricted access to professional dental care. Caregivers reported that residents typically receive only annual or biannual dental examinations from visiting dentists at the facility. Nevertheless, oral health concerns such as caries, gingivitis, or infections often remain unaddressed until symptoms become severe. When residents report toothaches, caregivers notify the responsible nursing staff, who then arrange for the residents to visit a dental clinic for further evaluation and management.

“If they have a toothache, we will inform the nurse, and they will arrange everything to go to the clinic.”

[P12]

“When there’s a program, then there’s a check-up for all the elderly, maybe once a year.”

[P9]

Although the primary aim of this study was to explore the barriers and challenges in providing oral care to older adults in a long-term care facility, caregivers also shared valuable suggestions that reflected important points to help improve the oral care provided by caregivers, counteracting the challenges outlined. The caregivers highlighted that what could result in a major improvement in the oral care practices provided, especially for dependent older adults, is better training and education on oral care, particularly for those with complex needs such as physical disabilities and older adults with gingivitis and prosthesis. The caregivers suggested that these training sessions should include hands-on demonstrations and practices for more effective training.

“Non-government organizations (NGOs) or other organizations could come here to teach the proper ways to care for the older adult’s teeth.”

[P1]

Moreover, caregivers also suggested increasing accessibility to domiciliary oral care by dental professionals. More frequent visits from dental clinics are needed for oral examinations to be performed, the early detection of oral diseases, and to maintain or improve the residents’ oral health.

“Perhaps there should be more frequent visits from clinics or agencies to conduct dental checkups for the residents.”

[P14]

However, they stressed that these domiciliary oral care visits should not be primarily for oral checkups but instead full comprehensive care, including scaling and restoration to address the residents’ oral health status. The caregivers recognized the need for the older adults in this facility to receive dental treatment but that there was a lack of support from dental professionals.

“If they have cavities, help us by filling their decayed teeth. Those with tartar buildup need scaling, so help us with that too.”

[P7]

The caregivers also stated that having a dedicated oral care team within the long-term care facility who could attend to the residents’ oral health on a consistent basis would be helpful. The caregiver explained that the residents’ oral care is often deprioritized in their daily care routine.

“I think there should be someone specifically for oral care in home because sometimes we overlook that part.”

[P13]

## 4. Discussion

The convergent mixed-methods design of this study allowed for our quantitative and qualitative findings to be triangulated to generate a more comprehensive understanding of the results. The high proportion of residents with fewer than 20 teeth and poor oral function identified through clinical assessments was reinforced by caregivers’ reports of chewing difficulties, oral pain, and neglected denture care. Similarly, while over 80% of residents required dentures, qualitative findings highlighted systemic barriers such as lack of caregiver training and limited access to professional dental services that explained why denture hygiene and usage were poor. The quantitative data on residents’ dependency levels also aligned with caregivers’ descriptions of varied oral care practices ranging from self-care among more independent residents to full assistance for those who are bedridden. Divergences were also informative; for instance, although dependency was quantitatively measured, qualitative insights revealed how cultural norms and behavioral resistance complicated caregiving beyond what numeric scores could capture. Together, these converging and complementary strands of evidence provide a more nuanced account of the oral health challenges faced in long-term care facilities.

This study provides new insights into the oral health status of older adults in a Malaysian public long-term care facility and highlights the unique challenges caregivers face in daily oral care. While similar issues have been documented globally [6], our findings highlight context-specific barriers shaped by cultural, systemic, and behavioral factors in Malaysia, thereby contributing novel evidence to the literature on institutional oral care in Southeast Asia.

Oral and denture hygiene among residents was found to be generally poor, and many sleep with dentures. This practice, also reported elsewhere [24], is especially concerning in the Malaysian context, where limited caregiver supervision at night may compound risks of oral ulcers, fungal infections, and aspiration pneumonia [25]. The low uptake of dentures despite significant tooth loss is another important finding. Prior studies have linked impaired masticatory function to frailty and nutritional decline [26,27]; however, our data suggests that cultural dietary habits may exacerbate this risk. In Malaysia, staple foods often include fibrous vegetables and tough-textured items such as meat, which are difficult to chew without functional dentition, potentially accelerating frailty trajectories.

The variability in oral care practices across residents reflected both functional differences and caregiver approaches. Residents capable of self-care maintained minimal routines such as brushing and rinsing, while dependent individuals relied heavily on caregiver assistance. Individualized oral hygiene plans, shown to improve outcomes elsewhere [28], remain underdeveloped in Malaysian LTCFs. This gap indicates the need for structured, locally adapted care protocols.

The caregivers reported that residents with complex medical conditions, particularly those experiencing xerostomia due to polypharmacy, showed accelerated oral decline. Xerostomia, prevalent in 30–40% of older adults, complicates oral hygiene and increases the risk of caries [29]. The Malaysian setting is notable because older adults frequently receive multiple medications for chronic conditions such as diabetes and hypertension, both highly prevalent nationally [30]. Polypharmacy-induced xerostomia places additional strain on caregivers, who often lack training to identify or manage such complications.

Behavioral resistance, such as refusal of care and aggression, emerged as a recurrent challenge, consistent with international findings on dementia-related care refusal [31]. In Malaysia, these behaviors are further shaped by intergenerational dynamics, as caregivers often struggle to persuade residents who perceive themselves as socially and hierarchically superior due to age. Such cultural norms of respect toward elders may inadvertently hinder assertive caregiving and contribute to inconsistent oral hygiene practices. At the same time, lifestyle factors such as smoking and vaping, commonly observed among residents, exacerbate oral disease risks and reflect a wider cultural acceptance of tobacco use among Malaysian older adults. These behavioral and cultural challenges are compounded by psychological conditions such as depression and anxiety, which are known to reduce self-care engagement [32], but these conditions are likely underdiagnosed in local LTCFs, further impeding effective oral care.

An important contribution of this study is its documentation of caregiver training gaps in the Malaysian LTCF context. The caregivers in our study reported reliance on experiential knowledge rather than formal instruction, resulting in inconsistent practices. Elsewhere, training interventions have demonstrated improved oral hygiene outcomes [33,34]. Our findings reinforce the need for culturally appropriate caregiver training in Malaysia, particularly strategies for managing resistant residents through behavioral and communication techniques.

Access to dental professionals was found to be a major challenge. While domiciliary oral care is sparse across many countries [6], the Malaysian context is marked by a limited number of dentists trained in geriatric oral care, and logistical barriers to transporting frail residents to dental clinics. Our findings highlight the urgency of integrating professional oral care into existing on-site medical care. This approach, complemented by interdisciplinary collaboration among dentists, nurses, and caregivers, could bridge the gaps in timely treatment and referral pathways.

System-level challenges, especially staff shortages, echo global concerns about caregiver burnout [35]. In Malaysia, this problem is intensified by reliance on publicly funded institutions with constrained budgets, which limit staff recruitment and training opportunities. When caregivers are overburdened, oral care is often deprioritized in favor of tasks perceived as more urgent, such as feeding or bathing the residents. Addressing this gap requires policy-level interventions that not only increase staffing but also ensure that oral health is recognized as an essential part of overall care. Measures such as designating oral health assistants within LTCFs and embedding oral care as a core component of caregiving duties could help distribute responsibilities more effectively and ensure that oral health does not remain a neglected aspect of institutional care.

### 4.1. Limitations

This study has several limitations that should be considered when interpreting the findings. First, its cross-sectional, descriptive design restricts causal inference; thus, the results should be viewed as descriptive associations rather than explanatory relationships. Second, the final sample size was slightly below the initial target, although a post hoc power analysis confirmed that the achieved sample retained acceptable power for descriptive purposes (β = 0.87). Third, cognitive function was not assessed with a validated screening tool. This decision was made to minimize participant burden and ensure ethical inclusion of only those able to provide informed consent, but this may limit comparability with studies that use formal cognitive assessments. Fourth, the qualitative findings of this study reflect the perspectives of a particular group of caregivers from a single facility; their views are likely influenced by their training, experience, and tolerance in managing residents who resist care, which may narrow the diversity of the perspectives. Finally, this study was conducted only in one location. However, it was a large, publicly funded nursing home with relatively strong institutional support and established links to government dental services. Despite having access to the above-mentioned resources, numerous challenges were still reported. It is therefore reasonable to assume that institutions with less support and fewer resources could face even greater barriers to providing adequate oral care for older adults.

### 4.2. Recommendations

Building on the challenges identified in this study, several practical and actionable recommendations are listed below that could guide policymakers and long-term care facility (LTCF) managers in strengthening oral health for older adults:

For Policymakers:

- Integrate oral health into aged-care standards: Establish oral health as a mandatory component in national LTCF guidelines and include oral health indicators in routine institutional audits.
- Expand domiciliary dental services: Invest in mobile dental units and allocate funding for on-site dental care, ensuring access for frail and dependent older adults.
- Develop geriatric dentistry capacity: Strengthen workforce training and create specialist pathways for dentists to deliver care in LTCFs.
- Secure dedicated funding: Provide budget allocations for oral health programs within LTCFs, including staffing support and training modules.

For LTCF Managers:

- Implement structured caregiver training: Introduce culturally adapted, hands-on training programs for oral care, with regular refreshers and embedded competencies in staff orientation.
- Establish oral care champions or assistants: Designate trained staff within each facility to oversee daily oral health routines, monitor compliance, and support colleagues.
- Adopt standardized protocols: Ensure consistent oral care practices by developing protocols for denture hygiene, management of resistant residents, and care for individuals with complex medical conditions.
- Facilitate interdisciplinary collaboration: Strengthen referral pathways and routine collaboration with dental clinics, nurses, and allied health professionals to manage oral health issues in a timely manner.

## 5. Conclusions

This study achieved its objective of assessing the oral health status of older adults in a long-term care facility and uncovering the challenges faced by caregivers. The findings reveal that poor oral and denture hygiene, high dependency, and inconsistent caregiver practices remain major barriers to adequate oral care. These insights provide actionable directions for policy and practice, including structured caregiver training, integration of oral health into aged-care standards, and expansion of domiciliary dental services to improve residents’ quality of life. Future research should expand upon these findings to include multiple facilities and evaluate the impact of training and on-site dental interventions, providing evidence to guide sustainable policies for integrating oral health into long-term care systems.

## Figures and Tables

**Table 1:** Characteristics of older adult residents (N = 115).

Variable	n	%	Mean	S.D
Gender				
Male	67	58.3		
Female	48	41.7		
Age			73	6.8
Race				
Malay	76	66.1		
Chinese	24	20.9		
Indian	13	11.3		
Other	2	1.7		
Level of Dependency				
No to low dependency	76	66.1		
Medium dependency	28	24.3		
High dependency	11	9.6		
Oral Frailty				
Less than 20 natural teeth remaining	106	92.2		
Difficulty eating tough food for past 6 months	61	53.0		
Choked on tea or soup recently	102	88.7		
Frequent dry mouth	83	72.2		
Articulatory oral motor skills (Fewer than 6 times per second)	110	95.7		
Risk of poor oral function	109	94.8		
Caries status				
Decayed (D)			1.0	2.4
Missing (M)			24.5	6.4
Filled (F)			0.3	1.0
Total DMFT			25.7	4.3
Percentage of caries experience (DMFT > 0)	115	100.0		
Plaque score (n = 67)			83.7	20.8
Dentition status				
Partially edentulous	67	58.3		
Fully edentulous	48	41.7		
Denture use				
Wear denture	21	18.3		
Need denture	94	81.7		
Denture Hygiene (n = 21)				
Upper			3.1	1.3
Lower			3.1	1.1

**Table 2:** Demographics of the caregivers.

Participant Identification	Age	Gender	Years of Experience as Caregiver
P1	39	Female	17
P2	26	Female	2
P3	53	Female	9
P4	29	Male	5
P5	34	Male	9
P6	33	Male	9
P7	27	Male	2
P8	27	Male	7
P9	28	Male	2
P10	30	Female	5
P11	21	Female	1
P12	19	Female	5
P13	35	Female	5
P14	38	Female	10
P15	26	Female	2
P16	24	Male	1

**Table 3:** Challenges in providing oral care for older adults in a long-term facility setting.

Theme	Subtheme	Number of Mentions
Health and Oral Health Conditions of Older Adults	Poor oral hygiene and maintenance	16
	Missing teeth	9
	Chewing difficulty	6
	Oral pain and discomfort	5
	Complex medical condition	3
Variety in Oral Care Practices	Self-cleaning of mouth	9
	Denture care	6
	Assisted oral care	8
Older Adults’ Attitudes and Behaviors	Uncooperative or lack of cooperation	16
	towards dental care	
	Smoking and vaping habits	8
System Factor	Lack of manpower	7
	Lack of caregivers’ knowledge or skills in oral care for older adults	8
	Lack of formal training on oral care for older adults among caregivers	16
	Limited access to professional dental care	8

## Data Availability

The data presented in this study are available on request from the corresponding author due to ethical reasons.

## Abbreviations

The following abbreviations are used in this manuscript:

FGD	Focus Group Discussion
S.D.	Standard Deviation
